# Soil and Seed

**Published:** 1902-06-07

**Authors:** 


### SOIL AND SEED.

In delivering the annual oration before the Medical Society Dr. Stephen Mackenzie chose for his theme a subject of great and of yearly increasing importance, namely, the powers of natural resistance to diseases of microbic origin. "The tendency of the medical mind at the present time," says Dr. Mackenzie, seems to be to attach too great importance to the germ or seed and too little to the body or soil in diseases of microbic origin." While by no means making light of the seed, a study of the soil and of the condition under which the natural body resists the attacks of disease-germs becomes a matter of the highest therapeutic importance, for in the treatment of such maladies the highest success seems to be attained by encouraging and reinforcing "natural resistance" rather than by any direct attacks we may be able to make upon the invading microbe. Turning to tuberculosis as an illustration, Dr. Mackenzie says that it is more than possible that the danger of infection from food has been over-estimated, and quotes Cattle as saying that it is not only milk that goes into the mouths of children; they grub about on all-fours, plastering their fingers with the tuberculous dust, and then put their fingers in their mouths; so that, quite independently of their food, they have plenty of opportunity of receiving tuberculous infection, even by way of the mouth; indeed, "we have learned that the bacillus tuberculosis is so widely disseminated that no one is exempt from the risk of infection." The interest would seem then at first sight to centre upon the means by which those of us who escape the disease have been protected rather than upon the manner in which those who succumb have been infected. The fact would seem to be, however, that, as Kanthack said, mankind is naturally resistant to tuberculosis, and that, starting from this basis, we have really to consider the artificial conditions by which man's natural resistance is turned to vulnerability. In regard to this Dr. Mackenzie appeals to experience, which shows that heredity, alcoholism, the occurrence of measles, whooping cough, and their accompanying broncho-pneumonia, may all act as predisponents to the disease, while practically our most successful treatment of such tuberculous diseases as are not accessible to the surgeon consists in increasing the vigour and resistance of the patient by life in the open air, abundance of sunlight, thorough ventilation of his apartments, abundant and nourishing food, and strict attention to general and personal hygiene. "In fact, our principal object and power, whether we recognise it or not, is to endeavour by every means in our hands to raise the natural resistance of the body to the microbe, to devote our attention to rendering the soil an unfavourable one to the growth of the tubercle bacillus." In reference to many of the measures which are now being advocated, he says it must be remembered that the striking diminution in the prevalence of consumption which can be shown to have taken place, not only in this but in some other countries, had been steadily going on before any effective measures had been taken for disinfecting or destroying sputum or separating the healthy from the sick; and that this improvement in the statistics regarding consumption has been going on even in spite of the great migration from the country to the towns which has been in progress during recent years, and has evidently "been due to improved sanitation, to better draining of the land, to improved disposal of sewage, and to the better housing, clothing, and feeding of the population." Thus the war against consumption becomes a matter not so much of the treatment of the individual, but of the rearing of "a population of more robust physique, offering a more resistant soil to the seeds of tuberculosis," an aim which, however, he confesses, may to some seem "transcendental, ideal, involving great economic and social problems." Turning now to rheumatic fever, which from recent observations appears to be very probably set up by microbic infection, Dr. Mackenzie says that, even if its bacterial nature should be fully established, "the patient work which has been done on the clinical side will not have been thrown away," for "we shall still have to take account of the soil no less than of the seed." The fact that some persons escape while others are attacked, although exposed to apparently the same influences, shows that an individual predisposition, as well as a microbic infection, is at work. Even in appendicitis, microbic as may be the origin of the inflammation, there are predisposing causes arising sometimes from preceding morbid conditions of the parts, by which their nutrition and consequently their resistance is lowered, sometimes from a general lessening of resisting power which would occasionally seem to run in families. Unfortunately in this disease this individual predisposition shows no sign of its existence until the occurrence of an attack, yet one cannot doubt that in appendicitis, as in other maladies which arise from microbic infection, the soil is quite as important a factor as the seed in the etiology of the disease.

